# Local Infections Associated with Ventricular Assist Devices: Materials-Related Challenges and Emerging Solutions

**DOI:** 10.3390/ma18194541

**Published:** 2025-09-30

**Authors:** Klaudia Cholewa, Przemysław Kurtyka, Agnieszka Szuber-Dynia, Artur Kapis, Maciej Gawlikowski

**Affiliations:** 1 Faculty of Biomedical Engineering, Silesian University of Technology, Roosevelta 40 Str., 41-800 Zabrze, Poland; agnieszka.szuber-dynia@polsl.pl (A.S.-D.); maciej.gawlikowski@polsl.pl (M.G.); 2 Institute of Heart Prostheses, Foundation of Cardiac Surgery Development, 345a Wolności Str., 41-800 Zabrze, Poland; pkurtyka@frk.pl (P.K.); akapis@frk.pl (A.K.)

**Keywords:** local infections, antibacterial materials, mechanical circulatory support, biocompatibility

## Abstract

Although heart transplantation remains the gold standard in the treatment of advanced heart failure, the limited availability of donor organs and the growing number of patients requiring long-term care have necessitated wider implementation of mechanical circulatory support (MCS). Ventricular assist devices (VADs) substantially improve survival and quality of life, yet their clinical use is still constrained by serious complications, most notably local infections at percutaneous exit sites. This challenge persists across all device generations, from extracorporeal pulsatile pumps to contemporary continuous-flow systems. While fourth-generation concepts based on transcutaneous energy transfer are under development, unresolved issues such as thermal tissue injury continue to impede their adoption. This review critically examines current evidence on local infections, with particular emphasis on the role of biomaterials in bacterial colonization. The clinical burden and microbial etiology, dominated by Staphylococcus aureus and Staphylococcus epidermidis, are outlined, together with the limitations of existing material solutions, which lack durable antimicrobial activity. These infections frequently result in tissue necrosis, sepsis, rehospitalization, and elevated treatment costs, and their management is further complicated by the global rise in antimicrobial resistance. By synthesizing available data and identifying key shortcomings of current materials, this review underscores the urgent need for next-generation biomaterials with enhanced biocompatibility, resistance to microbial adhesion, and intrinsic or functionalized antimicrobial activity. Such advances are essential to improve the long-term safety and clinical outcomes of MCS therapy.

## 1. Introduction

**Figure 1:**
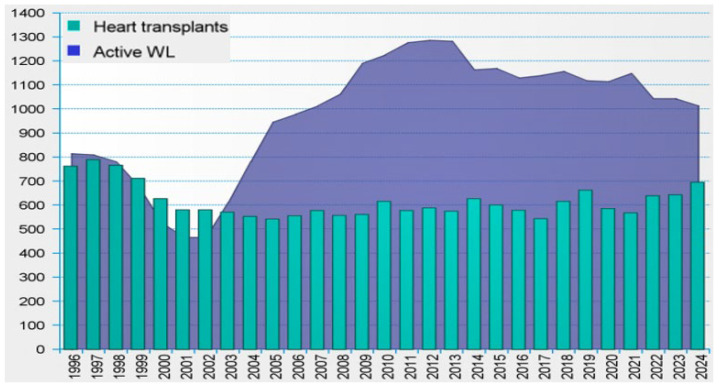
Number of heart transplants vs. active waiting list in the Eurotransplant (1996−2024) [1].

The growing incidence of heart failure, combined with the persistent shortage of donor organs, highlights the urgent need for effective alternatives to heart transplantation. Despite progress in medical and surgical therapies, transplantation remains limited by the availability of suitable donors and the complexity of both the procedure and post-transplant care. Although it continues to be the standard of care for patients with end-stage heart failure who are not responsive to conventional treatment, the number of transplants performed each year falls short of demand, leading to a continuous increase in the number of patients on waiting lists. This persistent mismatch between transplant demand and donor availability is clearly demonstrated in Figure 1 [1].

Progress in pharmacotherapy and the development of advanced mechanical circulatory support (MCS) technologies have contributed to a reduction in mortality among patients with advanced heart failure. MCS devices now serve not only as a bridge to transplantation or myocardial recovery but also as destination therapy, particularly through the use of implantable continuous-flow rotary blood pumps [2,3]. Based on device location (extracorporeal or intracorporeal) and the type of blood flow generated, ventricular assist device (VAD) systems are typically classified into three main generations.

First-generation MCS systems are extracorporeal devices that generate pulsatile blood flow using pneumatic or electric actuation to support cardiac output. These systems include a flexible internal membrane that separates the blood chamber from the actuation chamber. During the filling phase, negative pressure in the actuation chamber enables passive inflow of blood into the pump. In the ejection phase, the membrane is compressed by pneumatic or electric force, pushing blood out through the outflow cannula and into the arterial circulation. This cyclical movement produces a pulsatile flow pattern that partially mimics the native heartbeat. The pumps are typically connected to an external controller via a pneumatic or electrical conduit, allowing adjustment of flow parameters to meet individual hemodynamic needs [4]. For left ventricular support, the inflow cannula is inserted into the left atrium or the ventricular apex, while the outflow cannula is connected to the ascending aorta.

Second-generation VADs are implantable systems that provide continuous blood flow through the use of axial-flow rotary pumps supported by mechanical bearings. These devices are suitable not only as a bridge to transplantation but also as long-term destination therapy, as they do not require prolonged hospitalization, unlike first-generation pulsatile pumps [5]. The inflow cannula is positioned in the apex of the left ventricle, and the outflow graft is connected to the ascending aorta. The continuous-flow mechanism enables efficient unloading of the failing ventricle and restoration of systemic circulation with fewer moving parts and a smaller device profile.

Third-generation VADs are fully implantable continuous-flow systems that incorporate either active magnetic levitation or passive hydrodynamic suspension to stabilize the rotor without mechanical contact. These advanced bearing-free technologies allow for lower rotational speeds while maintaining effective blood flow, significantly reducing shear stress, hemolysis, and activation of prothrombotic pathways [6]. The absence of mechanical bearings enhances device durability and minimizes the risk of blood element damage, platelet activation, and coagulation cascade initiation [7]. As a result, third-generation devices offer improved long-term performance and reduced complication rates compared to earlier technologies.

**Figure 2:**
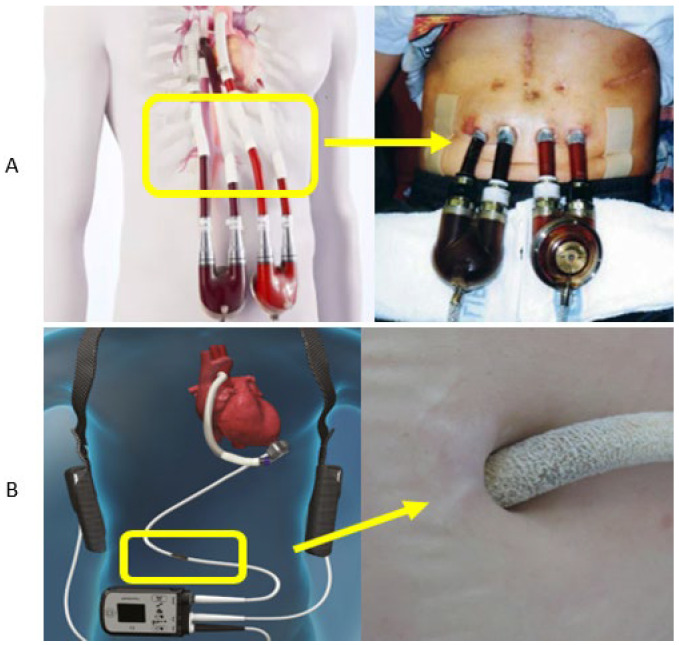
Schematic and clinical images of percutaneous access sites: (**A**) cannulas of a first-generation extracorporeal pulsatile pump (Thoratec Paracorporeal BiVAD) [12,13]; (**B**) driveline of an implantable continuous-flow rotary pump (HeartMate III) [9,14].

Despite the substantial clinical benefits of MCS systems across all generations, serious complications remain common. These include progressive right ventricular failure, bleeding events, and a high risk of local infections at percutaneous sites [8,9,10,11]. Figure 2 presents both schematic and clinical examples of percutaneous passages for cannulas and power cables in MCS devices [9,12,13,14]. In most cases, the cannulas and driveline exit the body through the abdominal wall via a skin-exit site, typically located in the upper abdominal quadrant [15,16].

Despite the introduction of newer generations of VADs and the implementation of various strategies to prevent perioperative infections, local infections still occur at a considerable rate, ranging from 18% to 59% among all patients receiving MCS therapy [8,9,10]. Notably, the risk and incidence of infection increase proportionally with the duration of support. Percutaneous infections at the driveline or cannula exit sites are considered device-specific infections [17,18] and represent the most common type of infection associated with VADs. These infections remain one of the greatest challenges in the clinical management of patients receiving MCS [19]. The issue of local infections surrounding the driveline or cannulas has been extensively discussed in the literature [20,21,22], yet no sufficiently effective solution has been developed to eliminate this complex clinical problem.

**Figure 3:**
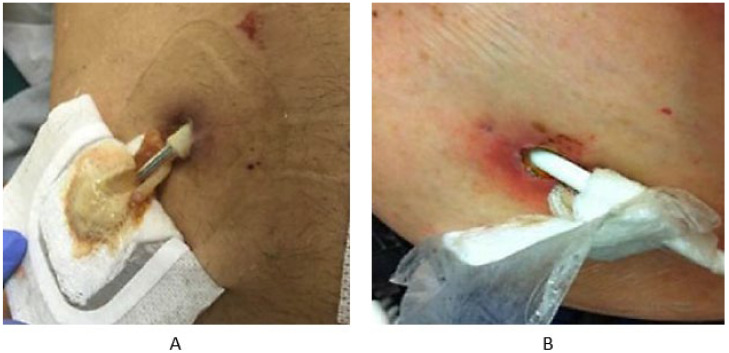
Representative images of complicated driveline infections: (**A**) visible purulent discharge from the driveline skin-exit site; (**B**) erythema surrounding the skin-exit site [8].

In the latest 2024 report by the Journal of Heart and Lung Transplantation, a revised classification system for percutaneous site infections was introduced, replacing the previous superficial vs. deep tissue distinction. The updated classification distinguishes between uncomplicated and complicated driveline infections, aiming to simplify clinical diagnosis. Key clinical indicators differentiating these categories include the presence of bacteremia, fungal or drug-resistant organisms, elevated local temperature, abscess formation, and erythema at the skin-exit site [9,23]. A driveline infection is classified as complicated when accompanied by a positive blood culture, confirmed internal infection along the subcutaneous tunnel of the driveline or cannula (detected via imaging), persistent fungal or drug-resistant pathogens, or the presence of fluid collections or abscess at the exit site (Figure 3) [8,23].

Infections at the percutaneous exit sites of cannulas or drivelines may present as localized, mild cases or may progress along the subcutaneous tunnel toward the pump pocket or bloodstream, leading to serious and potentially life threatening complications such as systemic infection, endocarditis, hemorrhagic or ischemic stroke, and sepsis-related mortality [23,24,25].

Advances in MCS engineering have not translated into a proportional decrease in infection burden. The nonreplaceable, percutaneous components continue to act as stable niches for colonization and biofilm formation, especially as time on support increases. Efforts to integrate existing data are constrained by variability in reporting practices. Even with the adoption of the uncomplicated versus complicated framework, many series lack standardized imaging of tunnel involvement, uniform microbiology reporting, and time dependent outcomes that allow comparison across centers and devices. Current preventive measures remain dominated by perioperative care bundles and local antisepsis. These strategies mitigate early events but do not address material and tissue mechanics, micromotion at the exit site, or durability of any antimicrobial effect over long support periods.

The aim of this review is to consolidate and critically assess current evidence on local infections at percutaneous exit sites in MCS, with emphasis on material-related antimicrobial approaches. This work constitutes a planned step within a broader research, which will proceed with structured in vitro and in vivo studies. The ultimate goal is to establish a solid preclinical basis for evaluating novel surface modifications intended to provide long-term antibacterial protection at the skin-device interface.

## 2. Materials Utilized at Percutaneous Exit Sites

Cannulas in first-generation MCS systems are polymeric conduits approved for blood contact. They are available in a range of diameters (typically 6 to 12 mm), depending on patient age and anatomical characteristics. The driveline, which transmits power and control signals, utilized in second- and third-generation VADs typically consists of a Teflon-polyurethane cable surrounded by a smooth medical-grade silicone jacket. To reduce friction between internal cable elements, biocompatible silicone oil is used. The driveline connects the implanted pump to the external controller and exits the body through the skin, necessitating the application of a tissue-integrated dressing or sewing cuff made of medical-grade fabric. These cuffs facilitate dermal tissue ingrowth, which supports the gradual replacement of fibrin-platelet clots with granulation tissue and, over time, collagen fibers [19]. This biological sealing mechanism helps isolate the exit site from the external environment and provides a protective barrier against microbial invasion.

The most commonly used medical materials for this purpose are velour patches made of Dacron (polyethylene terephthalate, PET). Dacron is a biocompatible polyester known for its high mechanical strength and chemical resistance, widely used in vascular grafts, cuffs, and surgical dressings [26,27]. In its woven or knitted velour form, it exhibits a fibrous, rough, and porous structure, with pore sizes ranging from tens to hundreds of micrometers. This topography increases the contact surface area between the implant and surrounding tissues, enhancing protein adsorption, cell migration, and collagen fiber penetration. As a result, the material promotes active biological integration, enabling fibroblasts and capillaries to infiltrate the porous matrix and establish a stable, long-term tissue interface [28,29]. Over time, the inter-fiber spaces become filled with connective tissue, reducing susceptibility to micromotions and bacterial colonization.

The driveline used in implantable VADs is approximately 100 cm long and consists of a smooth silicone sheath and a short external segment (~8 cm) covered with polyester or Dacron velour to enhance tissue integration and stabilize the skin-exit site. Traditionally, a portion of the velour segment is intentionally left outside the body, usually at least 2 cm, to promote local tissue growth and improve driveline stability, reducing the risk of displacement and trauma [29,30]. A more recent surgical technique involves tunneling the entire velour-covered segment subcutaneously, thereby creating a silicone-skin interface (SSI) at the exit site [31]. Medical-grade silicone, composed of polydimethylsiloxane (PDMS) elastomer, is widely used in percutaneous MCS applications due to its excellent biocompatibility, flexibility, and chemical inertness. It is generally well tolerated by biological tissues and tends to form a thin fibrous capsule without eliciting strong inflammatory responses. Its smooth, homogeneous, nonporous surface minimizes bacterial adhesion and biofilm formation, significantly lowering the risk of driveline-associated infections. Clinical studies have demonstrated that using a smooth silicone interface at the skin-exit site reduces local infection rates to approximately 1.7%, compared to over 20% in patients with exposed velour segments [28,29]. Retrospective analyses from multicenter SSI registries confirm the reduced infection incidence in patients managed with subcutaneous velour placement compared to those with traditional external velour exposure [29,30,32]. Due to its surface characteristics, silicone does not support active tissue ingrowth and cannot establish a strong mechanical integration with the skin. Although the smooth surface promotes hygiene and reduces friction-related trauma, it may compromise driveline anchorage and require additional surgical fixation techniques to ensure stability.

In extracorporeal MCS systems, the situation is more complex than in implantable devices. The cannulas used in these systems are significantly larger in both diameter and length compared to drivelines in implantable rotary pumps, which limits options for material innovation and surgical modification [33]. An additional challenge is the constant micromotion of the cannulas caused by pressure fluctuations during blood flow. In pulsatile extracorporeal pumps, the motion of the pump itself can also contribute to dynamic stress at the exit site, including transient changes in cannula diameter due to elasticity. The inherent stiffness of the cannula or driveline can lead to microtrauma at the skin-exit site, particularly during normal patient movement. These mechanical disruptions create vulnerable entry points for microbial colonization and increase the risk of infection.

All of the materials utilized in this field provide the structural stability and biocompatibility required for use at percutaneous exit sites, although, despite these advantages, they share the limitation of lacking intrinsic antibacterial activity, which facilitates bacterial adhesion and biofilm formation and thereby increases the risk of local infections. Studies on textiles have shown that Staphylococcus aureus, the pathogen most frequently associated with driveline infections, survives longer and proliferates more extensively on polyester fibers than on cotton, which has been linked to the material characteristics and higher surface roughness of polyester [34]. The selection of biomaterials alone cannot eliminate the infection risk, and surface modifications capable of providing durable antibacterial functionality must be prioritized. Without such innovations, even the biocompatible materials remain susceptible to biofilm formation and recurrent local infections, perpetuating one of the most significant unresolved complications of long-term circulatory support. Addressing these issues by developing effective surface modifications to improve the antimicrobial performance of these polymers should therefore be considered a key priority in the advancement of safer transcutaneous solutions.

## 3. Etiology and Diagnosis of Local Infections

### 3.1. Epidemiology of Local VAD Infections

The duration of VAD support is considered the most significant risk factor for driveline and cannula exit-site infections. As previously noted, infection risk increases steadily with longer support times [17,35,36,37]. This is largely due to prolonged exposure of the percutaneous interface to mechanical irritation or trauma, which compromises the skin barrier and promotes bacterial invasion. Data suggest the highest incidence of driveline infections occurs between 91 and 180 days post-implantation, significantly more than in the early postoperative period (within 90 days) [38].

Additional risk factors for exit-site infections include higher body mass index (BMI), diabetes mellitus, advanced age, chronic kidney disease, autoimmune conditions such as systemic lupus erythematosus and systemic sclerosis (scleroderma), as well as various dermatologic disorders [17,35,39]. In pediatric patients, younger age is sometimes noted as a risk factor due to longer support durations when VADs are used as a bridge to transplant rather than destination therapy [35]. According to the July 2023 INTERMACS report, patients with substance abuse histories (alcohol or drug use) are also at elevated risk for infection [40,41]. Inadequate hygiene and driveline care significantly contribute to infection risk, whereas consistent support from a partner or family caregiver has been associated with reduced infection incidence compared to rotating external nursing staff [17]. Although these risk factors are consistently reported, most available data derive from retrospective registry analyses or single-center observations, which introduces heterogeneity in definitions and follow-up. The relative contribution of individual comorbidities remains difficult to quantify, and causality cannot always be established. It should also be emphasized that non-medical factors, including socioeconomic status and access to structured caregiver support, are less frequently addressed in the literature despite their clear impact on outcomes.

### 3.2. Etiology of Local Infections

From a microbiological perspective, local infections are most often caused by Gram-positive bacteria, particularly Staphylococcus aureus and Staphylococcus epidermidis [9,42,43]. These organisms are known for biofilm formation, which increases their virulence, complicates treatment, and heightens the risk of bloodstream infection [9,44]. Biofilms limit the effectiveness of antibiotics even in the absence of formal antimicrobial resistance [45,46]. Reports from multiple centers indicate that 4% to 55% of *S. aureus* isolates are methicillin-resistant (MRSA), though methicillin-susceptible strains (MSSA) are also prevalent [14,43,47]. Other pathogens include Enterococcus, *Escherichia coli*, and various fungal organisms. Fungemia in patients with pulsatile MCS devices is associated with a mortality rate as high as 70–90% [14,47]. Early postoperative infections are more often linked to Staphylococcus species, whereas late infections are typically associated with environmental Gram-negative bacteria such as Pseudomonas aeruginosa, often related to moisture exposure in the home (e.g., bathing). A five-year single-center study showed that the same bacterial strain can persist at the exit site for months or even years, with later isolates genetically matching the initial strain, indicating long-term colonization [48]. These findings indicate that infections at driveline and cannula exit sites are not only frequent but also dominated by microorganisms with high capacity for persistence and recurrence. The ability of bacteria species to form biofilms, together with the emergence of methicillin resistance, illustrates why eradication is difficult despite optimized antibiotic regimens. The documented persistence of identical strains over prolonged periods emphasizes that exit-site infections should be considered chronic colonization processes rather than isolated acute events.

### 3.3. Biofilm Formation at Transcutaneous Exit Sites

Local infections at percutaneous exit sites are closely associated with the capacity of bacteria to adhere to cuff materials and initiate biofilm formation. The likelihood of biofilm development at percutaneous surfaces is determined not only by microbial factors but also by patient and device related conditions [49,50]. Prolonged implantation time, advanced age, diabetes, and immunosuppression are well-recognized risk factors that impair local defense mechanisms and facilitate microbial persistence. At the exit site, the presence of biological deposits such as fibrin or wound exudate creates a conditioning layer that enhances bacterial adhesion. Mechanical aspects, including moisture accumulation, minor skin trauma, and micromotion of the driveline cuff, further destabilize the barrier function of surrounding tissue. Equally important are the physicochemical characteristics of the cuff material, such as porosity, roughness, hydrophobicity, and surface energy, which govern the strength and stability of bacterial attachment [34,51]. Under favorable conditions, initial adhesion develops into structured communities that remain firmly integrated with the material surface. Biofilm alters the local microenvironment at the biomaterial-tissue interface and form a barrier that markedly reduces the effectiveness of antimicrobial penetration. Consequently, infections linked to biofilm-covered surfaces are significantly more difficult to eradicate than those caused by non-adherent bacteria [52]. In ventricular assist systems, this colonization constitutes both a microbiological concern and a material-related challenge, as the combination of host-related vulnerabilities and material properties directly influences the risk of persistent colonization and recurrent infection.

Qu et al. confirmed that biofilm formation occurs not only on the external driveline surface but also within subcutaneous tunnels, where progression is markedly accelerated. Their study highlighted that the characteristic structure of velour material, together with pores, micrographs and incomplete tissue integration observed in explanted drivelines, creates favorable conditions for sustained bacterial colonization. The authors further reported that biofilm frequently spreads along the driveline, linking superficial colonization with the high progression to deeper infections [50]. Adjusting the physicochemical properties of biomaterial surfaces has emerged as a complementary method to limit bacterial attachment and enhance long-term performance. By modifying parameters such as surface chemistry, charge, wettability, and micro- or nanotopography, it is possible to reduce protein adsorption and delay the cascade of events leading to biofilm formation.

### 3.4. Diagnostics Approaches

Diagnostic criteria for uncomplicated driveline infections include negative blood cultures, absence of systemic symptoms, no fluid collection or abscess on imaging, and clinical signs such as tenderness, pain, or erythema. Partial or full clinical response to antibiotic therapy further supports the diagnosis [23]. Microbiological cultures (blood and surface swabs) are essential to identify pathogens at the exit site. In addition to lab diagnostics, imaging such as ultrasonography and computed tomography (CT) is used. Ultrasound is preferred for identifying superficial fluid or abscess collections, while CT scans help evaluate the depth of infection [53]. Both modalities have limitations due to artifacts from metallic components [11].

Although recent definitions provide a more standardized framework for reporting driveline and device-related infections, significant diagnostic challenges remain. Differentiating superficial colonization from true deep tissue infection is still difficult, as exit-site cultures and local clinical signs often lack specificity.

## 4. Prevention and Management of Local Infections in MCS

**Figure 4:**
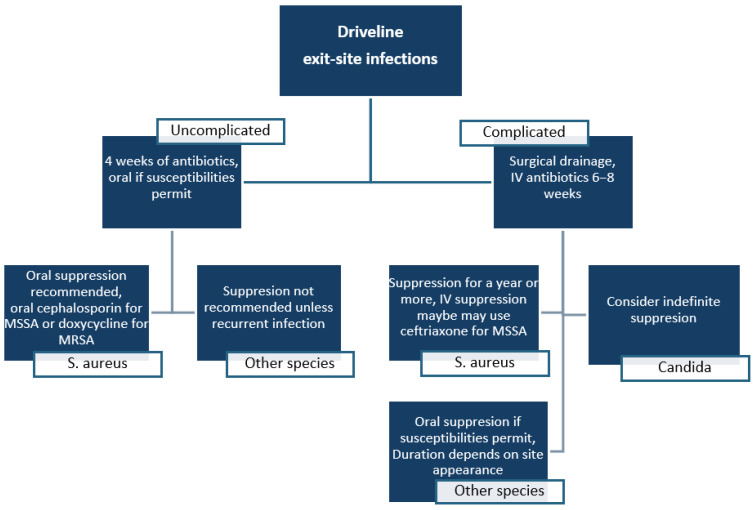
Management algorithm for percutaneous exit-site infections based on literature data [11].

Exit-site infections remain one of the most serious complications associated with VADs and require a multidisciplinary therapeutic approach (Figure 4). Uncomplicated infections (formerly classified as superficial) are generally managed with oral or intravenous antibiotics for several weeks, depending on the pathogen involved. In contrast, complicated (formerly deep tissue) infections typically require 6–8 weeks of intravenous therapy, often combined with surgical intervention or, in critical cases, device exchange [14].

For *S. aureus* infections, treatment strategy depends on the depth of infection and antibiotic susceptibility profile. The International Society for Heart and Lung Transplantation (ISHLT) recommends 2–4 weeks of oral or IV antibiotics for superficial infections. For MSSA, oral cephalosporins (first-generation) are typically used in suppressive therapy, while doxycycline is recommended for MRSA. IV treatment options for MSSA include nafcillin or cefazolin, provided susceptibility is confirmed. Vancomycin is the standard for MRSA, with linezolid as an alternative, particularly in resistant cases. In chronic or biofilm-associated infections, combination therapy including rifampin may be considered [14].

In cases of advanced infection caused by antibiotic-resistant pathogens, surgical reoperation is often required to debride the driveline and relocate its skin-exit site. Such procedures pose significant risks to the health and survival of patients with end-stage heart failure. In many instances, this necessitates the creation of a completely new subcutaneous tunnel for driveline repositioning, which further increases procedural complexity and patient risk.

Despite recent reports indicating partial effectiveness of both antibiotic regimens and surgical strategies, the overall management of driveline infections remains far from satisfactory. Increasing antimicrobial resistance limits the long-term success of pharmacological therapy, while surgical interventions inevitably expose patients to additional risks and often reduce quality of life by necessitating repeated procedures.

## 5. Economic Considerations

Local infections in patients implanted with left VADs represent not only a serious clinical threat but also a significant economic burden for healthcare systems. Clinically, these infections are associated with reduced quality of life and the need for prolonged treatment, including extended hospitalization and intensive antibiotic therapy. MCS-specific infections, particularly driveline exit-site infections, are strongly associated with increased rehospitalization rates and longer hospital stays [29]. According to 2021 data, the average cost of a single hospitalization due to such infections was £7662 in the United Kingdom and approximately $13,600 in the United States [54]. These differences reflect the variability in organizational and financial structures across national healthcare systems. Furthermore, the cost of managing driveline infections may vary depending on the severity of the infection, the need for surgical intervention, and the overall length of hospitalization.

**Figure 5:**
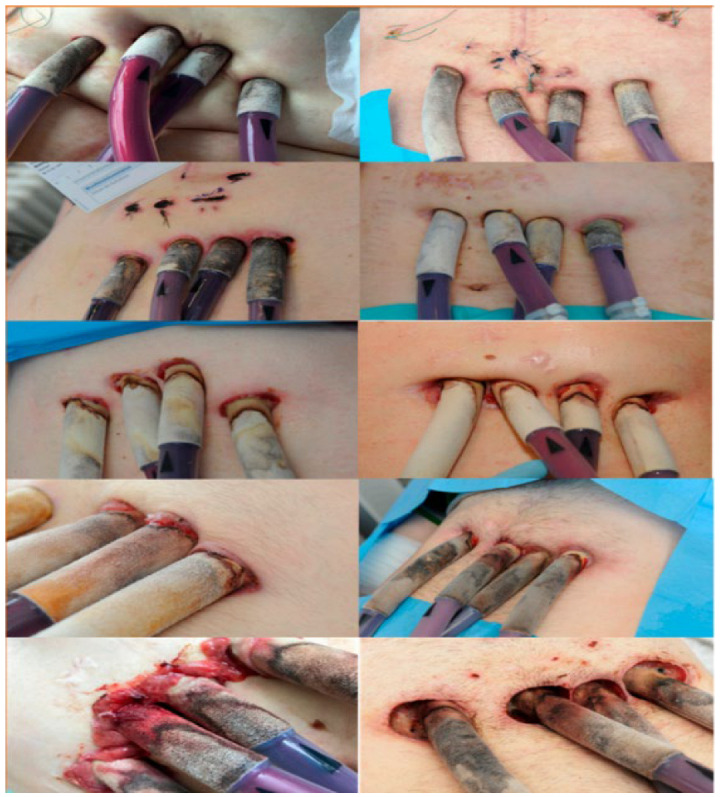
Stages of local infection progression in patients treated with BiVAD (biventricular mechanical circulatory support) [21].

Effective prevention and early diagnosis strategies are therefore essential not only for improving patient outcomes but also for reducing the economic strain on hospitals and healthcare infrastructures. The rising prevalence of antibiotic resistance further contributes to the growing costs of treating bacterial infections in patients receiving MCS therapy. The need for combination of antibiotic regimens or surgical reoperations adds additional financial pressure, which must be accepted to ensure patient survival. Figure 5 presents the clinical stages of localized infection development at the percutaneous exit site of cannulas in patients supported with extracorporeal pulsatile BiVADs [21]. Such infections negatively affect patients’ quality of life by increasing their sense of helplessness, dependence, and loss of control, resulting in both individual and systemic burdens [44]. These infections frequently require multiple hospitalizations, repeated follow-up visits, and the use of expensive medications and procedures, which also affect the psychosocial functioning of patients and their families.

The financial impact of driveline infections is widely acknowledged, yet available evaluations concentrate mainly on hospitalization and direct treatment costs. Limited consideration is given to expenses related to prolonged antibiotic therapy or the high demand on hospital staff resources. As a result, current estimates likely understate the overall economic burden, indicating the need for broader analyses that account for the full spectrum of associated costs.

## 6. Innovative Solutions and Future Directions

The growing issue of antibiotic resistance among pathogens has made infections associated with biomaterials utilized at transcutaneous exit sites a significant clinical challenge. This has led to an urgent need for innovative, durable antimicrobial strategies. Different approaches have been explored to limit the risk of local infections, including the incorporation of metallic elements with intrinsic antimicrobial properties, antibiotic-based strategies, and a variety of surface modification techniques. These concepts aim to reduce bacterial adhesion and slow down biofilm development on materials used at percutaneous interfaces, although their durability and long-term clinical effectiveness remain to be fully established.

### 6.1. Metal Nanoparticles

One of the most promising approaches involves surface functionalization using metal nanoparticles, such as silver, zinc oxide, copper or titanium, which exhibit broad-spectrum antimicrobial activity against both Gram-positive and Gram-negative bacteria [55,56,57].

Zinc oxide nanoparticles have been extensively investigated as antimicrobial surface modifiers for textiles and polymer surfaces for biomedical use. Recent studies have demonstrated that ZnO-coated cotton fabrics, manufactured either by sonochemical synthesis or plasma-assisted deposition techniques exhibit significant inhibitory effects against both Gram-positive and Gram-negative bacteria. Nabi et al. reported up to 89.5% reduction of *S. aureus* and 84.9% reduction of *E. coli* on ZnO-coated material, highlighting the potential of this approach in preventing bacterial colonization on porous, fibrous surfaces [58]. Similarly, Giedraitienė et al. showed that two-sided ZnO coatings deposited on cotton by low-temperature plasma provided durable antibacterial and antifungal activity, particularly against *Klebsiella pneumoniae* and *Candida albicans* [55]. From a biomedical engineering and clinical perspective, such findings are highly relevant to percutaneous device applications, where porous surfaces create niches favorable to microbial persistence. However, despite their broad-spectrum activity and relative biocompatibility, ZnO coatings present notable limitations. The antimicrobial efficacy can vary depending on coating thickness, particle size and deposition method [55,58,59,60]. Given the risks of non-uniform nanoparticle distribution and potential agglomeration, comprehensive studies are still needed to evaluate durability, stability, and overall biocompatibility of the coatings in long-term in vivo applications.

Silver remains a highly valued antimicrobial element in the modification of porous biomaterials, particularly medical textiles and wound dressings. Numerous experimental studies have demonstrated that silver nanoparticle coatings confer broad-spectrum activity against clinically relevant pathogens such as *S. aureus*, *E. coli*, and *Klebsiella pneumoniae*. Durable silver layers deposited on cotton fabrics achieved bacterial reductions exceeding 90% while maintaining key properties, including mechanical strength [61,62]. Ag–PTFE nanocomposite coatings developed for urinary catheters inhibited biofilm formation by more than 90% for *S. aureus* and *E. coli*, while preserving host cell viability, further underscoring their potential for application in long-term implantable and percutaneous devices [63]. In vitro evaluations have shown that the antibacterial performance of Ag–PTFE is closely linked to silver-ion release, which is initially high but declines after several days before reaching a stable level, which may reduce long-term efficacy [63]. Recent research highlights that the antibacterial performance of porous biomaterials containing silver is counterbalanced by risks of local cytotoxicity. In an ex vivo porcine skin model, silver dressings penetrated dermal tissue and induced oxidative stress, DNA damage, decreased keratinocyte and fibroblast viability, and increased IL-6 release [64]. These adverse effects were shown to depend not only on the presence of silver, but also on its chemical form, the specific formulation, and the applied concentration.

Copper has emerged as another promising antimicrobial surface treatment of porous biomaterials [56,65]. Sputtered copper layers deposited on PTFE demonstrated significant bactericidal effects against *S. epidermidis* and *E. coli*, with the strongest inhibition observed for non-annealed coatings [56]. In contrast, thermal annealing reduces efficacy due to oxidation and diminished ion release. Studies on medical textiles coated with cuprous oxide nanoparticles reported nearly complete suppression of biofilm formation of *S. aureus*, *E. coli*, MRSA, and multidrug-resistant *E. coli*, with reductions frequently exceeding 99% [66]. These findings highlight the capacity of copper nanostructures to impair both sensitive and resistant pathogens on polymeric and textile substrates, but their performance is highly dependent on coating stability, the chemical form of copper, and processing conditions, which can markedly influence antimicrobial efficacy.

Titanium dioxide nanoparticles are highly stable, biocompatible, and exhibit intrinsic antibacterial activity that is only weakly dependent on bacterial species, making them broadly effective against both Gram-positive and Gram-negative pathogens [67,68]. Higher antimicrobial efficacy is associated with smaller particle size and specific structural characteristics, such as amorphous forms or doped compositions [67]. Studies on textile polyester composites incorporating titanium dioxide nanoparticles reported bacterial growth reductions often exceeding 95–99%, with silver doping further extending efficacy and durability [57,67]. Recent research further demonstrate that titanium dioxide textile coatings can achieve bacterial growth reduction exceeding 99% against *S. aureus* and *E. coli*, underscoring their strong potential for practical use in medical textiles [68,69]. However, inhalation of nanoscale titanium dioxide has been associated in toxicological studies with pulmonary inflammation and potential carcinogenicity, which raises occupational safety issues during material processing and necessitates careful risk assessment for large-scale biomedical use [70,71]. These factors indicate that while titanium dioxide coatings are promising, their practical translation requires optimization of activation mechanisms, spectrum of antimicrobial action, and safe handling protocols.

Although coatings based on metal elements have demonstrated encouraging antimicrobial effects in experimental and textile-based applications, none of these approaches currently provide a reliable solution for local infections in MCS. Their antibacterial activity is often short-lived, highly dependent on ion release kinetics, surface processing, or external activation, and accompanied by risks of cytotoxicity or limited biocompatibility. Importantly, driveline cannulas and percutaneous components cannot be routinely exchanged, which makes long-term stability and durable protection against biofilm formation essential. As such, existing coatings remain unsuitable for clinical translation in this context. Further research will therefore focus on developing novel antimicrobial strategies that combine safety, durability, and effective prevention of microbial colonization, potentially integrating these elemental coatings into optimized multifunctional systems. In the longer term, the ultimate goal remains the elimination of transcutaneous components altogether through the advancement of fully implantable circulatory support systems [44].

### 6.2. Antibiotics Solutions

Approach that may be effective in addressing local infections is the use of antibiotic-eluting coatings. This strategy has been investigated mainly in vascular surgery, where Dacron and ePTFE grafts have been modified with antibiotics such as rifampicin, vancomycin, ceftriaxone, and gentamicin. Antibiotic-loaded gelatin sealants on Dacron inhibited bacterial growth while maintaining graft performance, with vancomycin-containing variants showing the most consistent effect [72]. Also experiments with gentamicin-coated ePTFE grafts showed reduced bacterial adherence and a sustained antibacterial effect over an extended period, with lipid-based gentamicin formulations achieving the most favorable balance between antimicrobial performance and tissue compatibility [73].

Alongside these strategies, advanced wound dressings have been developed with the aim of providing localized antimicrobial protection. Smart dressings incorporate antibiotics, including aminoglycosides (gentamicin, streptomycin), β-lactams (ampicillin, cefazolin, ceftazidime), glycopeptides (vancomycin, teicoplanin), fluoroquinolones (ciprofloxacin, levofloxacin), tetracyclines (doxycycline, minocycline), and sulfonamides (sulfadiazine, sulfamethoxazole) into polymeric matrices, where they act locally through controlled release mechanisms [74]. In addition to conventional antibiotics, antimicrobial peptides have been integrated into hydrogels and nanofiber systems, in some cases combined with photosensitive molecules to enhance antibacterial activity and biofilm disruption. Such approaches enable targeted antimicrobial delivery, reduce the reliance on systemic therapy, and contribute to lowering the risk of resistance development. The therapeutic efficacy of antibacterial wound dressings that deliver antibiotics is governed by the release kinetics of the drug as well as the physicochemical properties of both the active compound and the polymeric carrier. These solutions face important limitations, including the relatively short duration of drug release, challenges in achieving uniform coating and reproducible performance, and the scarcity of robust clinical evidence confirming their long-term effectiveness.

### 6.3. Antibacterial Peptides

Antimicrobial peptides are increasingly investigated as functional coatings for biomaterials because they can be integrated directly into the surface design rather than relying on systemic drug delivery. From a materials engineering perspective, two principal strategies are applied. The first involves covalent immobilization of antimicrobial peptides onto polymer or metallic substrates to create active, durable surface layers [75]. This approach ensures long-term stability, minimizes the risk of peptide leaching, and provides continuous antibacterial protection at the material-tissue interface. Immobilization may limit the spectrum of activity to surface-contacting bacteria and raises engineering challenges in maintaining peptide conformation and bioactivity after binding. The second strategy incorporates antibacterial peptides within hydrogel or polymer matrices, enabling localized and controlled release through tunable material properties such as hydrophilicity, degree of crosslinking, and responsiveness to environmental triggers [76]. Such systems can provide a sustained antimicrobial effect that extends beyond the immediate surface, allowing diffusion into surrounding tissues and addressing early colonization more effectively. These modifications also face limitations, including potential loss of activity during prolonged release and the need to balance drug loading with biocompatibility and mechanical stability of the carrier.

Although a broad spectrum of antimicrobial concepts has demonstrated efficacy in diverse biomedical settings, their implementation in local infections associated with MCS poses unique challenges. Cannulas and drivelines constitute permanent percutaneous components, and their non-exchangeable nature necessitates antibacterial strategies that ensure sustained activity and structural resilience over extended periods. As a result, approaches characterized by transient release kinetics or insufficient coating stability, while attractive in other clinical contexts, are of limited relevance in this field. To provide a structured overview, the main advantages and limitations of the most relevant strategies are summarized in Table 1.

The comparison emphasizes that although several antimicrobial strategies demonstrate promising activity, none currently provide a durable solution for driveline infections. In future studies, both the evaluation of individual approaches and the exploration of their combined application will be undertaken to identify long-term and clinically viable solutions for preventing device-related infections.

## 7. Shared Challenges in Other Clinical Applications

The challenges of percutaneous device-related infections are not unique to MCS. Patients with chronic conditions that require permanent transcutaneous access are similarly affected, including those undergoing peritoneal dialysis, long-term hemodialysis through tunneled central venous catheters, or urinary drainage via nephrostomy tubes. In each of these scenarios, complete elimination of skin-penetrating components is currently unfeasible, and exit sites represent persistent points of vulnerability to microbial colonization and biofilm formation. In peritoneal dialysis, infections of the Tenckhoff catheter exit site and tunnel are among the most frequent causes of peritonitis, often necessitating catheter removal and transition to hemodialysis. Infections associated with tunneled central venous catheters remain a leading cause of morbidity and hospital readmission in long-term hemodialysis patients, despite standardized care reports and antiseptic protocols [77,78]. Nephrostomy tubes, while essential for urinary tract decompression in obstructive disease, are also well recognized for their susceptibility to recurrent local infections and subsequent urosepsis [79].

The consequences of such infections extend beyond immediate clinical risk. Management typically requires prolonged systemic antibiotic therapy, repeated hospitalizations, and, in many cases, replacement of the affected device. These interventions not only compromise therapeutic continuity but also impose a heavy psychological toll on patients, contributing to diminished quality of life, dependency, and loss of autonomy. From a healthcare system perspective, the cumulative burden is substantial: costs related to infection management include antimicrobial treatment, surgical or interventional procedures for device replacement, and prolonged inpatient care [79].

Taken together, these challenges highlight that percutaneous infections represent a cross-disciplinary problem. Advances that will be made in the prevention of driveline infections in MCS may offer valuable insights and potential translational solutions for other medical domains in which permanent percutaneous devices remain indispensable.

## 8. Conclusions

Infections at percutaneous exit sites remain one of the most critical barriers to long-term success in MCS. Despite considerable progress in the engineering of VADs and related technologies, driveline and cannula infections continue to represent a major source of morbidity and mortality. Conventional preventive strategies can reduce infection rates but have not eliminated the problem. The permanent nature of percutaneous components makes these devices particularly vulnerable, since once implanted they cannot be replaced or exchanged, and the biological challenge of maintaining a stable interface with the skin persists throughout the duration of support.

Research over the past decade has explored a wide spectrum of material-based approaches intended to limit bacterial adhesion and biofilm formation. Metallic nanoparticles have been incorporated into coatings to provide broad-spectrum antimicrobial activity, although concerns remain regarding cytotoxicity, aggregation, and the durability of their antibacterial effect. Antibiotic-eluting solutions have demonstrated efficacy in controlled laboratory conditions, but they are often constrained by rapid drug release, risk of resistance, and poor long-term stability. Advanced wound dressings designed for localized antibiotic release have shown promise in wound care and could be adapted for driveline management, though their effectiveness in a high motion, moisture-exposed interface remains unproven. Antimicrobial peptides, whether covalently immobilized on surfaces or embedded in hydrogels for controlled release, provide another attractive avenue, but challenges with stability, activity retention, and large-scale clinical validation limit their current applicability. Strategies that rely on surface functionalization offer potentially long-lasting modifications, yet their translation into clinically robust systems is still at an early stage.

The available evidence indicates that no single strategy has yet achieved the combination of mechanical durability, biocompatibility, and long-term antimicrobial activity required for safe use in MCS systems. Many promising solutions remain at the stage of in vitro testing or small animal studies, with little evidence of sustained benefit under clinically realistic conditions. The development of future biomaterials for MCS must therefore focus on multifunctionality: combining anti-adhesive and antimicrobial effects, ensuring mechanical stability under micromotion, and preserving tissue integration and patient safety over years of support.

Fully implantable systems without percutaneous components may provide a definitive solution by eliminating the chronic breach of the skin barrier. Until such systems are clinically feasible, however, the refinement of surface-engineered, biofunctional materials remains the most promising strategy for reducing the burden of local infections and improving the long-term outcomes of patients dependent on MCS devices.

This review represents an initial step in addressing the problem of driveline-associated infections and provides the groundwork for subsequent research. Upcoming investigations will focus on identifying antibacterial strategies tailored to materials utilized at transcutaneous exit sites. Their effectiveness and biocompatibility will be evaluated through systematic in vitro analyses and complemented by preclinical in vivo studies, with the aim of establishing clinically applicable solutions.

## Figures and Tables

**Table 1:** Summary of antimicrobial approaches with potential applicability to transcutaneous exit-sites [55,56,57,58,59,60,61,62,63,64,65,66,67,68,69,70,71,72,73,74,75,76].

Material/ Strategy	Mechanism	Advantages	Limitations
Zinc oxide (ZnO)	Zn^2+^ ions and reactive oxygen species damage bacterial membranes and cellular components	Effective against Gram-positive, Gram-negative bacteria and fungi, cost-effective	Efficacy varies with coating thickness, particle size, deposition method, long-term durability unclear
Silver (Ag)	Ag^+^ ions disrupt bacterial membranes, proteins and DNA, leading to membrane permeability disruption	Broad-spectrum activity, long-used in biomaterials	Cytotoxicity concerns, diminishing ion release over time, coating stability challenges
Copper (Cu)	Cu^2+^ ions rapidly inactivates bacteria and damage bacterial membranes and enzymes	Strong activity including against resistant strains, cost-effective	Coating stability issues, potential cytotoxicity depending on formulation
Titanium dioxide (TiO_2_)	TiO_2_ produces reactive oxygen species under light exposure that damage bacterial membranes and biomolecules, inhibiting growth and biofilm formation	Biocompatible, stable, provide durable coatings on textiles and polymers	Safety concerns with nanoparticle inhalation
Antibiotic-eluting coatings	Controlled diffusion of antibiotic from surface into surrounding tissue, creating a local concentration that inhibits bacterial adhesion and early biofilm formation	Provides targeted antimicrobial effect at the biomaterial surface, reduces systemic exposure, allows adaptation to different antibiotics depending on expected pathogens	Short drug release duration, potential resistance, uneven coating, coating stability under mechanical stress remains uncertain
Smart dressings	Antibiotic or peptide release from dressing matrix upon skin-material contact, sometimes triggered by local environmental changes such as pH, enzymes, or moisture, enabling on-demand antimicrobial activity	Localized, responsive antimicrobial delivery, versatile antibiotic options, reduced need for systemic therapy, possibility of integrating monitoring or sensing functions	Limited evidence from long-term clinical use, mechanical stability and drug release reproducibility under real-life conditions remain challenging
Antibacterial peptides	Disrupt bacterial membranes, causing leakage of cellular contents and cell death, or penetrate intact membranes and interfere with intracellular processes by binding to nucleic acids or proteins.	Dual strategy (surface-immobilized contact-active and hydrogel carriers), low resistance risk	Immobilization limits effect to contact zones, release systems may lose activity, mechanical stability needed

## Data Availability

No new data were created or analyzed in this study.

## References

[1] Eurotransplant Statistics. https://statistics.eurotransplant.org/.

[2] Roesel M.J., Nersesian G., Neuber S., Thau H., Wolff von Gudenberg R., Lanmueller P., Hennig F., Falk V., Potapov E., Knosalla C. (2024). LVAD as a Bridge to Transplantation—Current Status and Future Perspectives. Rev. Cardiovasc. Med..

[3] Han J.J., Acker M.A., Atluri P. (2018). Left Ventricular Assist Devices. Circulation.

[4] Wu E.L., Stevens M.C., Pauls J.P., Steinseifer U. (2018). First-generation ventricular assist devices. Mechanical Circulatory and Respiratory Support.

[5] Kuehl M., Garbade J. (2017). The evolution of left ventricular assist devices—A moment to reflect. J. Thorac. Dis..

[6] Tu J., Xu L., Li F., Dong N. (2024). Developments and Challenges in Durable Ventricular Assist Device Technology: A Comprehensive Review with a Focus on Advancements in China. J. Cardiovasc. Dev. Dis..

[7] Kozik D., Alsoufi B. (2023). Pediatric Mechanical Circulatory Support—A Review. Indian J. Thorac. Cardiovasc. Surg..

[8] Hernandez G.A., Nunez Breton J.D., Chaparro S.V. (2017). Driveline Infection in Ventricular Assist Devices and Its Implication in the Present Era of Destination Therapy. Open J. Cardiovasc. Surg..

[9] Qu Y., Peleg A.Y., McGiffin D. (2021). Ventricular Assist Device-Specific Infections. J. Clin. Med..

[10] Juraszek A., Smólski M., Kołsut P., Pięta A., Gutmajster E., Pająk K., Kuśmierczyk M. (2021). Prevalence and Management of Driveline Infections in Mechanical Circulatory Support—A Single Center Analysis. J. Cardiothorac. Surg..

[11] Aburjania N., Hay C., Sohail M. (2021). Continuous-flow left ventricular assist device systems infections: Current outcomes and management strategies. Ann. Cardiothorac. Surg..

[12] https://www.openpr.de/news/1206728/Berlin-Heart-gibt-erste-Implantation-einer-Ueberbrueckungsloesung-bei-Patienten-mit-Einkammerherz-bekannt.html.

[13] Oosterom A., Jonge N., Kirkels J.H., Rodermans B., Sukkel E., Klöpping C., Ramjankhan F., Lahpor J.R. (2007). End-stage heart failure and mechanical circulatory support: Feasibility of discharge from hospital. Neth. Heart J..

[14] Zinoviev R., Lippincott C.K., Keller S.C., Gilotra N.A. (2020). In Full Flow: Left Ventricular Assist Device Infections in the Modern Era. Open Forum Infect. Dis..

[15] Balestra N., Fredericks S., Vieira Caetano da Silva A., Rodrigues R.C.M., Nunes D.P., Pedrosa R.B.S. (2023). Driveline dressings used in heartmate patients and local complications: A retrospective cohort. Heart Lung.

[16] Molina E.J., Shah P., Kiernan M.S., Cornwell W.K., Copeland H., Takeda K., Fernandez F.G., Badhwar V., Habib R.H., Jacobs J.P. (2021). The Society of Thoracic Surgeons Intermacs 2020 Annual Report. Ann. Thorac. Surg..

[17] Dettbarn E., Prenga M., Stein J., Müller M., Hoermandinger C., Schoenrath F., Falk V., Potapov E., Mulzer J., Knierim J. (2024). Driveline infections in left ventricular assist devices—Incidence, epidemiology, and staging proposal. Artif. Organs.

[18] Kusne S., Mooney M., Danziger-Isakov L., Kaan A., Lund L.H., Lyster H., Wieselthaler G., Aslam S., Cagliostro B., Chen J. (2017). An ISHLT consensus document for prevention and management strategies for mechanical circulatory support infection. J. Heart Lung Transplant..

[19] McCandless S.P., Ledford I.D., Mason N.O., Alharethi R., Rasmusson B.Y., Budge D., Stoker S.L., Clayson S.E., Doty J.R., Thomsen G.E. (2015). Comparing velour versus silicone interfaces at the driveline exit site of HeartMate II devices: Infection rates, histopathology, and ultrastructural aspects. Cardiovasc. Pathol..

[20] Tan Z., Zeng L. (2020). Post-operative infection in mechanical circulatory support patients. Ann. Transl. Med..

[21] Kremer J., El-Dor A., Rivinius R., Schlegel P., Sommer W., Warnecke G., Karck M., Ruhparwar A., Meyer A.L. (2022). Wound Infections in Adult Patients after Berlin Heart^®^ EXCOR Biventricular Assist Device Implantation. Life.

[22] Eckmann C., Sunderkötter C., Becker K., Grabein B., Hagel S., Hanses F., Wichmann D., Thalhammer F. (2024). Left Ventricular Assist Device-Associated Driveline Infections as a Specific Form of Complicated Skin and Soft Tissue Infection/Acute Bacterial Skin and Skin Structure Infection—Issues and Therapeutic Options. Curr. Opin. Infect. Dis..

[23] Aslam S., Cowger J., Shah P., Stosor V., Copeland H., Reed A., Morales D., Giblin G., Mathew J., Morrissey O. (2024). The International Society for Heart and Lung Transplantation (ISHLT): 2024 Infection Definitions for Durable and Acute Mechanical Circulatory Support Devices. J. Heart Lung Transplant..

[24] Haglund N.A., Davis M.E., Tricarico N.M., Keebler M.E., Maltais S. (2015). Readmissions after Continuous Flow Left Ventricular Assist Device Implantation: Differences Observed Between Two Contemporary Device Types. ASAIO J..

[25] Zierer A., Melby S.J., Voeller R.K., Guthrie T.J., Ewald G.A., Shelton K., Pasque M.K., Moon M.R., Damiano R.J., Moazami N. (2007). Late Onset Driveline Infections: The Achilles’ Heel of Prolonged Left Ventricular Assist Device Support. Ann. Thorac. Surg..

[26] Rubinfeld G., Levine J.P., Reyentovich A., DeAnda A., Balsam L.B. (2015). Management of Rapidly Ascending Driveline Tunnel Infection. J. Card. Surg..

[27] Jaquiss R.D.B., Imamura M., Guleserian K.J. (2010). Berlin Heart Implantation for Congenital Heart Defects. Oper. Tech. Thorac. Cardiovasc. Surg..

[28] Stulak J.M., Davis M.E., Haglund N., Dunlay S., Cowger J., Shah P., Pagani F.D., Aaronson K.D., Maltais S. (2016). Adverse events in contemporary continuous-flow left ventricular assist devices: A multi-institutional comparison shows significant differences. J. Thorac. Cardiovasc. Surg..

[29] Dean D., Kallel F., Ewald G.A., Tatooles A., Sheridan B.C., Brewer R.J., Akhter S.A. (2015). Reduction in driveline infection rates: Results from the HeartMate II Multicenter Driveline Silicone Skin Interface (SSI) Registry. J. Heart Lung Transplant..

[30] Pelz G.B., Hashmi Z.A., Moraca R.J., Murali S., Benza R.L., Sokos G.G., Magovern G.J., Stutz S.L., Bailey S.H., Dean D.A. (2010). Battling the Achilles’ Heel of Left Ventricular Assist Devices: A Novel Technique to Reduce Drive Line Infections. J. Heart Lung Transplant..

[31] Camboni D., Zerdzitzki M., Hirt S., Tandler R., Weyand M., Schmid C. (2016). Reduction of INCOR^®^ driveline infection rate with silicone at the driveline exit site. Interact. Cardiovasc. Thorac. Surg..

[32] Ledford I.D., Miller D.V., Mason N.O., Alharethi R.A., Rasmusson B.Y., Budge D., Stoker S.L., Clayson S.E., Doty J.R., Thomsen G.E. (2011). Differential infection rates between velour versus silicone interface at the HeartMate II driveline exit site: Structural and ultrastructural insight into possible causes. J. Heart Lung Transplant..

[33] Horn M.V., Shah P., Menteer J., Herrington C., Guadiz D., Dechant D., Szmuszkovicz J. (2016). Cannula Site Management Strategies in Pediatric Ventricular Assist Device (VAD) Patients. J. Heart Lung Transplant..

[34] Jekiel K., Syguła-Cholewińska J. (2024). Assessment of different detection methods in bacteria survival on cotton and polyester textiles. J. Nat. Fibers.

[35] Martin S.I. (2013). Infectious Complications of Mechanical Circulatory Support (MCS) Devices. Curr. Infect. Dis. Rep..

[36] Goldstein D.J., Naftel D., Holman W., Bellumkonda L., Pamboukian S.V., Pagani F.D., Kirklin J. (2012). Continuous-flow devices and percutaneous site infections: Clinical outcomes. J. Heart Lung Transplant..

[37] Hannan M.M., Xie R., Cowger J., Schueler S., de By T., Dipchand A.I., Chu V.H., Cantor R.S., Koval C.E., Krabatsch T. (2019). Epidemiology of infection in mechanical circulatory support: A global analysis from the ISHLT Mechanically Assisted Circulatory Support Registry. J. Heart Lung Transplant..

[38] Zhou S., Yang G., Zhang M., Pienta M., Chenoweth C.E., Pagani F.D., Aaronson K.D., Fetters M.D., Chandanabhumma P.P., Cabrera L. (2023). Michigan Congestive Heart Failure Investigators. Mortality following durable left ventricular assist device implantation by timing and type of first infection. J. Thorac. Cardiovasc. Surg..

[39] Monkowski D.H., Axelrod P., Fekete T., Hollander T., Furukawa S., Samuel R. (2007). Infections associated with ventricular assist devices: Epidemiology and effect on prognosis after transplantation. Transpl. Infect. Dis..

[40] Jorde U.P., Saeed O., Koehl D., Morris A.A., Wood K.L., Meyer D.M., Cantor R., Jacobs J.P., Kirklin J.K., Pagani F.D. (2024). The Society of Thoracic Surgeons Intermacs 2023 Annual Report: Focus on Magnetically Levitated Devices. Ann. Thorac. Surg..

[41] Cogswell R., Smith E., Hamel A., Bauman L., Herr A., Duval S., John R., Roman D., Adatya S., Colvin-Adams M. (2014). Substance abuse at the time of left ventricular assist device implantation is associated with increased mortality. J. Heart Lung Transplant..

[42] Tattevin P., Flécher E., Auffret V., Leclercq C., Boulé S., Vincentelli A., Dambrin C., Delmas C., Barandon L., Veniard V. (2019). Risk factors and prognostic impact of left ventricular assist device-associated infections. Am. Heart J..

[43] Kamat I., Lamba H., Hines-Munson C., Hudson S., Liao K., Muldrew K.L., Green S., Terwilliger A., Kaplan H.B., Ramig R.F. (2022). Identifying causative microorganisms in left ventricular assist device infections as a guide for developing bacteriophage therapy. J. Surg. Res..

[44] Iyengar A., Feinman J., Jiang J., Song C., Kim S., Mathew A., Golec S., Rao A., Radakrishnan A., Asher M. (2025). Epidemiology and impact of device-specific infections on patients receiving left ventricular assist devices. JHLT Open.

[45] Leuck A.M. (2015). Left ventricular assist device driveline infections: Recent advances and future goals. J. Thorac. Dis..

[46] Skalweit M.J. (2018). Left Ventricular Assist Device Infections. Advanced Concepts in Endocarditis.

[47] Pieri M., Agracheva N., Fumagalli L., Greco T., De Bonis M., Calabrese M.C., Rossodivita A., Zangrillo A., Pappalardo F. (2013). I Infections Occurring in Adult Patients Receiving Mechanical Circulatory Support: The Two-Year Experience of an Italian National Referral Tertiary Care Center. Med. Intensiva.

[48] Pitton M., Valente L.G., Oberhaensli S., Casanova C., Sendi P., Schnegg B., Jakob S.M., Cameron D.R., Que Y.-A., Fürholz M. (2023). Dynamics of bacterial pathogens at the driveline exit site in patients with ventricular assist devices: A prospective, observational, single-center cohort study. J. Heart Lung Transplant..

[49] Damyanova T., Paunova-Krasteva T. (2025). What we still don’t know about biofilms—Current overview and key research information. Microbiol. Res..

[50] Qu Y., McGiffin D., Kure C., Ozcelik B., Fraser J., Thissen H., Peleg A.Y. (2020). Biofilm formation and migration on ventricular assist device drivelines. J. Thorac. Cardiovasc. Surg..

[51] Qu Y., McGiffin D., Hayward C., McLean J., Duncan C., Robson D., Kure C., Shen R., Williams H., Mayo S. (2020). Characterization of infected, explanted ventricular assist device drivelines: The role of biofilms and microgaps in the driveline tunnel. J. Heart Lung Transplant..

[52] Asma S.T., Imre K., Morar A., Herman V., Acaroz U., Mukhtar H., Arslan-Acaroz D., Shah S.R.A., Gerlach R. (2022). An overview of biofilm formation-combating strategies and mechanisms of action of antibiofilm agents. Life.

[53] Hupe J., Worthmann H., Ravenberg K.K., Grosse G.M., Ernst J., Haverich A., Bengel F.M., Weissenborn K., Schmitto J.D., Hanke J.S. (2023). Interplay between driveline infection, vessel wall inflammation, cerebrovascular events and mortality in patients with left ventricular assist device. Sci. Rep..

[54] Schueler S., Silvestry S.C., Cotts W.G., Slaughter M.S., Levy W.C., Cheng R.K., Beckman J.A., Villinger J., Ismyrloglou E., Tsintzos S.I. (2021). Cost-effectiveness of left ventricular assist devices as destination therapy in the United Kingdom. ESC Heart Fail..

[55] Giedraitienė A., Ružauskas M., Šiugždinienė R., Tučkutė S., Grigonis K., Milčius D. (2024). ZnO nanoparticles enhance the antimicrobial properties of two-sided-coated cotton textile. Nanomaterials.

[56] Lacmanova V., Nguyenova H.Y., Ulbrich P., Slepicka P., Sajdl P., Svorcik V., Reznickova A. (2020). Copper layers sputtered on PTFE: Effect of annealing on antibacterial performance. Mater. Today Commun..

[57] Prorokova N., Kumeeva T., Kholodkov I. (2020). Formation of Coatings Based on Titanium Dioxide Nanosolson Polyester Fibre Materials. Coatings.

[58] Nabi M., Hussain M.I.U., Shanaz S., Hussain S.A., Hussain I., Bhat M.A., ul Tarfain N., Kashoo Z.A., Badroo G.A., Hassan M.N. (2024). Antibacterial effectiveness of cotton textiles coated with zinc oxide nanoparticles fabricated using the sonochemical technique. Int. J. Res. Agron..

[59] Verbič A., Gorjanc M., Simončič B. (2019). Zinc Oxide for Functional Textile Coatings: Recent Advances. Coatings.

[60] Kachare K., Shendage S., Matwal S., Walvekar M., Vhanbatte S., Chang J.-Y., Ghule A. (2024). Bio-mediated synthesized zinc oxide coated on cotton fabric for antibacterial and wound healing application. Surf. Coat. Technol..

[61] Dumas L., de Souza M.C., Bonafe E.G., Martins A.F., Monteiro J.P. (2024). Optimized incorporation of silver nanoparticles onto cotton fabric using k-carrageenan coatings for enhanced antimicrobial properties. ACS Appl. Bio Mater..

[62] Rashid S., Ali M., Islam S., Iqbal M.O., Al-Rawi M.B.A., Naseem M. (2025). Enhancing the Antibacterial Properties of Silver Particles Coated Cotton Bandages Followed by Natural Extracted Dye. J. Ind. Text..

[63] Zhang S., Wang L., Liang X., Vorstius J., Keatch R., Corner G., Nabi G., Davidson F., Gadd G.M., Zhao Q. (2019). Enhanced Antibacterial and Antiadhesive Activities of Silver-PTFE Nanocomposite Coating for Urinary Catheters. ACS Biomater. Sci. Eng..

[64] Nešporová K., Pavlík V., Šafránková B., Vágnerová H., Odráška P., Žídek O., Císařová N., Skoroplyas S., Kubala L., Velebný V. (2020). Effects of wound dressings containing silver on skin and immune cells. Sci. Rep..

[65] Kudzin M.H., Kaczmarek A., Mrozińska Z., Olczyk J. (2020). Deposition of Copper on Polyester Knitwear Fibers by a Magnetron Sputtering System. Physical Properties and Evaluation of Antimicrobial Response of New Multi-Functional Composite Materials. Appl. Sci..

[66] Gupta A., Maruthapandi M., Das P., Saravanan A., Jacobi G., Natan M., Banin E., Luong J.H.T., Gedanken A. (2022). Cuprous Oxide Nanoparticles Decorated Fabric Materials with Anti-Biofilm Properties. ACS Appl. Bio Mater..

[67] Serov D.A., Gritsaeva A.V., Yanbaev F.M., Simakin A.V., Gudkov S.V. (2024). Review of Antimicrobial Properties of Titanium Dioxide Nanoparticles. Int. J. Mol. Sci..

[68] Rashid M.M., Simončič B., Tomšič B. (2021). Recent advances in TiO_2_-functionalized textile surfaces. Surf. Interfaces.

[69] Salama K.F., AlJindan R., Alfadhel A., Akhtar S., Al-Suhaimi E.A. (2024). Enhanced Antimicrobial Performance of Textiles Coated with TiO_2_ Nanoparticles. J. Ind. Text..

[70] Wang J., Fan Y. (2014). Lung Toxicity of Inhaled Titanium Dioxide Nanoparticles. J. Appl. Toxicol..

[71] Wolf S., Sriram K., Camassa L.M.A., Pathak D., Bing H.L., Mohr B., Samulin Erdem J. (2024). Systematic Review of Mechanistic Evidence for TiO_2_ Nanoparticle-Induced Lung Carcinogenicity. Nanotoxicology.

[72] Zhuravleva I.Y., Shadanov A.A., Surovtseva M.A., Vaver A.A., Samoylova L.M., Vladimirov S.V., Timchenko T.P., Kim I.I., Poveshchenko O.V. (2024). Which Gelatin and Antibiotic Should Be Chosen to Seal a Woven Vascular Graft?. Int. J. Mol. Sci..

[73] Lazić I., Obermeier A., Dietmair B., Kempf W.E., Busch A., Tübel J., Schneider J., von Eisenhart-Rothe R., Biberthaler P., Burgkart R. (2022). Treatment of Vascular Graft Infections: Gentamicin-Coated ePTFE Grafts Reveal Strong Antibacterial Properties In Vitro. J. Mater. Sci. Mater. Med..

[74] Cristea A.-G., Lisă E.-L., Iacob S., Dragostin I., Ștefan C.S., Fulga I., Anghel A.M., Dragan M., Morariu I.D., Dragostin O.-M. (2025). Antimicrobial Smart Dressings for Combating Antibiotic Resistance in Wound Care. Pharmaceuticals.

[75] Costa F., Carvalho I.F., Montelaro R.C., Gomes P., Martins M.C. (2011). Covalent Immobilization of Antimicrobial Peptides (AMPs) onto Biomaterial Surfaces. Acta Biomater..

[76] Mukhopadhyay S., Youssef S.H., Song Y., Nayak U.Y., Garg S. (2025). Harnessing the Power of Antimicrobial Peptides: From Mechanisms to Delivery Optimization for Topical Infections. Antibiotics.

[77] Bach N., Chi T.T.K., Trung L.C., Ngoc N.H.B., Hoa T.P.T., Huynh T.N.P., Khai T.H., Dao T.A., Tuoc P.X., Dung V.Q. (2025). Prevalence, Microbiology, and Outcome of Peritonitis in Peritoneal Dialysis Patients in Vietnam: A Multicenter Study. BMC Nephrol..

[78] Hajji M., Neji M., Agrebi S., Ben Nessira S., Ben Hamida F., Barbouch S., Harzallah A., Abderrahim E. (2022). Incidence and Challenges in Management of Hemodialysis Catheter-Related Infections. Sci. Rep..

[79] Delistefani F., Wallbach M., Müller G.A., Koziolek M.J., Grupp C. (2019). Risk factors for catheter-related infections in patients receiving permanent dialysis catheter. BMC Nephrol..

